# Lung resistance-related protein as a predictor of clinical outcome in advanced testicular germ-cell tumours

**DOI:** 10.1038/sj.bjc.6600803

**Published:** 2003-03-18

**Authors:** A J Zurita, J E Diestra, E Condom, X García del Muro, G L Scheffer, R J Scheper, J Pérez, J R Germà-Lluch, M A Izquierdo

**Affiliations:** 1 Department of Medical Oncology and Laboratory of Translational Research, Institut Català d'Oncologia, Av Gran Via Km 2.7, 08907 Barcelona, Spain; 2 Department of Pathology, Ciutat Sanitària i Universitària de Bellvitge, Feixa Llarga s/n, 08907 Barcelona, Spain; 3 Department of Pathology, Free University Hospital, De Boelelaan 1117, 1081 HV Amsterdam, the Netherlands

**Keywords:** testicular germ-cell tumours, lung resistance-related protein, P-glycoprotein, multidrug resistance-associated protein 1, breast cancer resistance protein, multidrug resistance

## Abstract

This study was undertaken to investigate the expression and predictive value for outcome of multidrug resistance-associated (MDR) proteins P-glycoprotein (Pgp), MRP1, BCRP, and LRP, in advanced testicular germ-cell tumours (TGCT). Paraffin-embedded sections from 56 previously untreated patients with metastatic TGCT were immunostained for Pgp, MRP1, BCRP, and LRP. All patients received platinum-based chemotherapy after orchidectomy. Immunostaining was related to clinicopathological parameters, response to chemotherapy, and outcome. Strong and intermediate expressions of the different MDR-related proteins were: 27 and 41% (Pgp), 54 and 37% (MRP1), 86 and 7% (BCRP), and 14 and 29% (LRP). P-glycoprotein and MRP1 associated, respectively, to low AFP (*P*=0.026) and high LDH levels (*P*=0.014), whereas LRP expression associated with high *β*-hCG levels (*P*=0.003) and stage IV tumours (*P*=0.029). No correlation was found between Pgp, MRP1, and BCRP expression and response to chemotherapy and survival. In contrast, patients with LRP-positive tumours (strong or intermediate expression) had shorter progression-free (*P*=0.0006) and overall survival (*P*=0.0116) than LRP-negative patients, even after individual log-rank adjustments by statistically associated variables. Our data suggest that a positive LRP immunostaining at the time of diagnosis in metastatic TGCT is associated with an adverse clinical outcome.

Current use of cisplatin-based chemotherapy and surgery in the first-line treatment of advanced testicular germ-cell tumours (TGCT) results in long-term disease-free status in about 80% of cases (Bosl and Motzer, 1997; Horwich *et al*, 1998). As a result of the high cure rate and the morbidity of treatment, clinical research has focused on optimising the management of this disease, tailoring therapies according to the risk of failure of the individual patient (Bosl and Motzer, 1997; Sonneveld *et al*, 2001). Factors associated with a poor-outcome have been analysed in several large studies, and include the extent of metastatic disease and serum levels of the *β*-subunit of human chorionic gonadotropin (*β*-hCG), *α*-fetoprotein (AFP) and lactate dehydrogenase (LDH) (Germà-Lluch *et al*, 1980; Mead *et al*, 1992). The most recent and commonly employed prognostic model for disseminated disease is the International Germ Cell Consensus Classification (IGCCC), which predicts relapse at 5 years for 12, 25, and 59% patients in the good, intermediate, and poor prognosis groups, respectively (International Germ Cell Cancer Collaborative Group, 1997).

Treatment failure in this disease is closely related to inherent or developed resistance to chemotherapy (Bosl and Motzer, 1997). Patients with refractory disease, failing to respond to initial treatment, have a worse prognosis (Motzer *et al*, 1991). However, current knowledge of the molecular determinants of chemoresistance involved in TGCT is only marginal (Houldsworth *et al*, 1998; Rao *et al*, 1998; Kersemaekers *et al*, 2002).

In particular, little is known about clinical multidrug resistance (MDR) in TGCT (Van Brussel and Mickisch, 1998). Multidrug resistance consists of *de novo* or acquired cross-resistance to structurally and functionally unrelated drugs, some of them relevant in the treatment of TGCT such as *Vinca* alkaloids, epipodophyllotoxins and anthracyclines (Lehnert, 1996). Among the various molecular mechanisms associated with MDR in experimental tumour models, one of the most extensively studied involves decreased drug accumulation due to enhanced efflux by ATP-dependent transporter proteins such as P-glycoprotein (Pgp), the human multidrug resistance-associated protein 1 (MRP1), and the recently described breast cancer resistance protein (BCRP). P-glycoprotein overexpression is associated with resistance to natural product drugs, including anthracyclines, etoposide, vincristine and vinblastine, and paclitaxel (Germann, 1996). Multidrug resistance-associated protein 1 is overexpressed in many non-Pgp-mediated MDR cell lines, conferring *in vitro* resistance, among others, to etoposide, vincristine, vinblastine, and to methotrexate after short-term exposure (Borst *et al*, 2000). Breast cancer resistance protein is a newly described transporter, isolated from mitoxantrone-selected MDR cell lines not expressing Pgp or MRP (Allikmets *et al*, 1998; Doyle *et al*, 1998). Breast cancer resistance protein is associated with cross-resistance to dauno- and doxorubicin, mitoxantrone, and camptothecins (Maliepaard *et al*, 1999).

Another protein related to an MDR-phenotype is the lung resistance-related protein (LRP) (Scheper *et al*, 1993). Lung resistance-related protein has been identified as the human major vault protein (MVP), the principal constituent of the complex ribonucleoprotein particles known as vaults (Scheffer *et al*, 1995). Of interest in TGCT, LRP/vaults have been associated with *in vitro* resistance to drugs such as etoposide, doxorubicin, vincristine, and paclitaxel, but also to nonclassical MDR drugs such as cis-platin and carboplatin (Izquierdo *et al*, 1996a; Kitazono *et al*, 1999). Although the function of vaults in both normal and cancer cells is not fully elucidated, they are thought to be involved in intracellular redistribution of drugs, reducing exposure of nuclear targets from cytotoxic agents (Chugani *et al*, 1993; Kitazono *et al*, 1999).

We undertook the present study to assess the immunohistochemical expression of the MDR-related proteins Pgp, MRP1, BCRP, and LRP in advanced TGCT, and to determine whether such expression is related to response to first-line chemotherapy and survival.

## PATIENTS AND METHODS

The expression of MDR-related proteins Pgp, MRP1, BCRP, and LRP was studied in paraffin-embedded samples from patients with advanced TGCT. This expression was retrospectively correlated with clinical and pathological characteristics of the patients, response to chemotherapy, and outcome.

### Sample selection

Between March 1990 and February 2001, 65 patients with primary advanced TGCT were treated in our institution. We studied banked samples from 56 specimens, corresponding to previously untreated patients undergoing orchidectomy for diagnostic evaluation and treatment. Samples were obtained from the Department of Pathology at the Ciutat Sanitària i Universitària de Bellvitge, Barcelona, Spain. Eligible patients were those with advanced disease (stage I excluded) undergoing induction chemotherapy, and with available material. The histological diagnosis was based on conventional morphologic examination of paraffin sections, following World Health Organization criteria (Mostofi and Sesterhenn, 1998).

### Treatment, evaluation of response, and survival

All patients were primarily treated with inguinal orchidectomy and postoperative cisplatin-based induction chemotherapy. Immediately after conventional induction chemotherapy, seven poor-risk patients (IGCCC) received high-dose chemotherapy as consolidation treatment. Complete responders to chemotherapy (CR) were patients with normalisation of serum tumour markers and no clinical or radiological evidence of residual masses, or absence of viable cancer cells such as seminoma, embryonal carcinoma (EC), immature teratoma, yolk sac tumour (YS), choriocarcinoma (CC), and syncytiotrophoblastic cells in completely resected residual masses (mature teratoma (MT) was not considered malignant component). Complete responders to chemotherapy plus surgery (CR-S) were those with viable malignant cells in completely resected residual masses. Incomplete responders (IR) were patients with persisting elevation of tumour markers, incomplete surgical resection of residual disease, or disease progression while receiving chemotherapy or within 1 month after the completion of chemotherapy. Events in the progression-free survival (PFS) analysis were: incomplete response, and relapse (rising tumour markers and/or an increase in tumour volume, unless caused by completely resectable MT). Patients in progression were managed with a variety of second-line chemotherapy regimens and surgery if appropriate. Only deaths related to TGCT progression were considered as events for overall survival (OS) analysis.

### Monoclonal antibodies

The IgG1 murine monoclonal antibody (MAb) JSB-1 was used for Pgp detection in a concentration of 50 *μ*g ml^−1^ in phosphate-buffered saline (PBS) plus 1% bovine serum albumin (BSA, Sigma, St Louis, MO, USA). For MRP1, the IgG2a MAb MRPm5 was used in a concentration of 5 *μ*g ml^−1^ in 1% PBS/BSA. For BCRP, the newly described IgG2a MAb BXP-21 was used in a concentration of 25 *μ*g ml^−1^ in 1% PBS/BSA. JSB-1, MRPm5, and BXP-21 were obtained from the Department of Pathology, Free University Hospital, Amsterdam, the Netherlands (Scheper *et al*, 1988; Flens *et al*, 1994; Maliepaard *et al*, 2001). None of these three MAbs cross react with the other MDR proteins (Maliepaard *et al*, 2001). Finally, the IgG1 murine MAb LRP (Transduction Labs, Los Angeles, CA, USA) was used for detection of LRP in a concentration of 2.5 *μ*g ml^−1^ in 1% PBS/BSA.

### Immunohistochemistry

Immunohistochemistry was performed on 4 *μ*m formalin-fixed, paraffin-embedded sections mounted on poly-L-lysin-coated glass microslides, and dried at 37°C O/N. The immunoperoxidase reaction conditions used for each MAb were selected on the basis of previous optimisation of the immunohistochemical protocols. After conventional deparaffination and rehydration, endogenous peroxidase activity was quenched by incubation in 3% H_2_O_2_ (10 min) at room temperature. Pretreatment in a pressure cooker (20 min) with either citrate buffer (10 mM citric acid pH 6.0 in distilled water) for MRPm5, LRP, and BXP-21, or EDTA (1 mM) for JSB-1 was performed to unmask epitopes. Next, samples were incubated (30 min) in normal rabbit serum (1 : 50 in 1% PBS/BSA), and then with the optimally diluted specific antibody (60 min) at room temperature in a humidified chamber. The MAb BXP-21 was detected by the Dako EnVision™+System-horseradish peroxidase (Dako Corp., Glostrup, Denmark) for 30 min (Diestra *et al*, 2002). A rabbit anti-mouse biotin-conjugated (1 : 150 for 30 min, Zymed Labs, San Francisco, CA, USA) followed by a streptavidin – horseradish peroxidase (1 : 500 for 1 h, Zymed) method was used for MAbs MRPm5 and LRP. JSB-1 was developed by a twice-each alternating incubation with a rabbit anti-mouse biotin-conjugated method (1 : 500 for 30 min, Dako) followed by a streptavidin–biotin complex (1 : 200 for 1 h, Dako). Staining with an irrelevant IgG or an isotype-specific antibody was routinely performed as a negative control procedure in the normal tissues and in all the clinical samples. Bound peroxidase was developed with 3,3′-diaminobenzidine (Sigma).

As positive control tissues for the immunohistochemical assays, liver was used for JSB-1 (Van der Valk *et al*, 1990), colon for MRPm5 (Flens *et al*, 1996) and LRP (Izquierdo *et al*, 1996b), and placenta for BXP-21 (Maliepaard *et al*, 2001). All slides were examined and scored by two independent observers (EC and AZ) blinded to the clinical data. Tumour cells were identified on morphological criteria. The MDR proteins were studied in adjacent slides from the most representative paraffin block available for each specimen. Only viable malignant germ cells (excluding MT elements) were considered for evaluation. By prior agreement, in accordance with previous literature, sections were evaluated in a semi quantitative way taking into account the percentage and intensity of staining of tumour cells (Baldini *et al*, 1995; Izquierdo *et al*, 1995; Arts *et al*, 1999; Eid *et al*, 2000). Three staining categories were established: negative (no staining), intermediate (positive staining in ⩽10% of tumour cells or weak and diffuse positive staining), and strong (moderate/strong staining in >10% of tumour cells). *A priori*, it was planned that the intermediate category needed to be grouped with either the negative or the strong category for statistical correlations (see below). The reasons for this were: (1) to ascertain whether the intermediate category provided more predictive information for each protein when grouped with any of the other two categories; (2) to perform meaningful statistical analysis with only two groups because of the limited number of patients; (3) a clinical relevant cut-off level of expression of MDR proteins is not well defined yet and it may be different for each protein and each tumour type. Intensity and pattern of staining on the different histological subtypes in each sample were recorded separately.

### Statistical analysis

Clinicopathological characteristics were determined previous to chemotherapy. These characteristics and response rates to induction chemotherapy were retrospectively assessed for their relation with Pgp, MRP1, BCRP, and LRP expression, using *χ*^2^ or the Fisher's exact two-tailed test as appropriate. For survival analysis, follow-up started the date of the first cycle of induction chemotherapy. Univariate survival analysis curves were generated using the Kaplan – Meier method and compared using the log-rank test (Kaplan and Meier, 1958; Mantel, 1966). A stratified Mantel – Haenszel test was performed to compare survival curves in order to adjust for the potential confounding effect of statistically associated variables upon differences between groups. Hypotheses were evaluated at a two-sided significance level of 0.05. The SPSS package (SPSS Inc, Chicago, IL, USA) was used for calculations.

## RESULTS

### Clinical and pathological characteristics, response to chemotherapy, and outcome

The median age at initial orchidectomy was 24 years (range, 16–56 years). No patient had a previous history of cancer, except for one (2%) having a contralateral and successfully treated TGCT more than 9 years before. The characteristics of the 56 patients included in the study are summarised in Table 1

**Table 1:** Clinical and pathological characteristics according to Pgp, MRP1, BCRP, and LRP expression

	**No. (%)**	**Pgp+(%)^a^**	**MRP1+(%)^a^**	**BCRP+(%)^a^**	**LRP+(%)^a^**
All cases	56(1 0 0)	15(27)	30(54)	48(86)	24(43)
*Histology*					
Nonseminoma	52(93)	13(25)	27(52)	46(88.5)	23(44)
Seminoma	4(7)	2(50)	3(75)	2(50)	1(25)
*Stage (Royal Marsden)*					
IM+II–III	39(70)	12(31)	20(51)	32(82)	13(33)
IV	17(30)	3(18)	10(59)	16(94)	11(65)***
*Pre-CT serum TM*					
AFP					
⩽1000 ng ml^−1^	45(80)	15(33)*	23(51)	39(87)	19(42)
>1000 ng ml^−1^	11(20)	0(0)	7(64)	9(82)	5(45.5)
β-hCG					
⩽5000 IU l^−1^	47(84)	13(28)	24(51)	40(85)	16(34)
>5000 IU l^−1^	9(16)	2(22)	6(67)	8(89)	8(89)****
LDH					
⩽1.5 upper N thresh	46(82)	13(28)	21(46)	39(85)	19(41)
>1.5 upper N thresh	10(18)	2(20)	9(90)**	9(90)	5(50)
*IGCCC*					
Good	35(62.5)	11(31)	16(46)	30(86)	13(37)
Intermediate or poor	21(37.5)	4(19)	14(67)	18(86)	11(52)
*Vascular invasion*					
Yes	38(68)	12(32)	22(58)	34(89)	17(45)
No	18(32)	3(17)	8(44)	14(78)	7(39)

CT=chemotherapy, N thresh=normal threshold, TM=tumour markers.

a Pgp+, MRP1+, and BCRP+ represent immunohistochemical category strong; LRP+ represents the grouping of categories intermediate and strong (see Patients and Methods, and Results).

* *P*=0.026 (Fisher's exact test).

** *P*=0.014 (Fisher's exact test).

*** *P*=0.029 (χ^2^ test).

**** *P*=0.003 (Fisher's exact test).

(left column). The 52 non-seminomatous TGCT included: 41 mixed tumours, seven EC, three YS, and one CC. Thirty-five (62%) were good-risk patients, 11 (20%) intermediate-risk, and 10 (18%) poor-risk, according to the IGCCC criteria (International Germ Cell Cancer Collaborative Group, 1997). Chemotherapy regimens used (Germà-Lluch *et al*, 1999) are listed in Table 2

**Table 2:** First-line chemotherapy regimens

	**Patients (*N*=56)**	
**Regimens**	**No.**	**%**
BEP^a^	37	66
BOMP-EPI	16	29
EP	2	4
Other platinum based	1	1
High dose^b^	7	13

BEP=etoposide, cisplatin, bleomycin, EP=etoposide, cisplatin, BOMP-EPI=bleomycin, vincristine, methotrexate, cisplatin/etoposide, ifosfamide, cisplatin.

a Includes one patient nonassessable for response.

b Immediately after conventional induction chemotherapy, seven poor-risk patients (IGCCC) received high-dose chemotherapy as consolidation treatment.

. Median number of cycles was 4 (range, 2–8). One patient (2%) was excluded from the analysis of response to chemotherapy because of a nonrelated-to-cancer death during treatment. Therefore, 55 patients were evaluable for tumour response to induction chemotherapy. Fifty-one CR (93%), three CR-S (5%), and one IR (2%) to first-line chemotherapy were accounted. Among those achieving a CR, 16 underwent surgery of residual masses: six showed either necrosis or fibrosis and 10 MT only. One of the CR-S patients actually progressed to first-line chemotherapy but underwent complete salvage surgery and continues relapse-free with more than 4 years of follow-up.

The median follow-up was 49.5 months (range 2–111 months). Seven patients (12.5%) progressed to first-line treatment, including 1 IR and 6 relapses. Four patients (7%) died of TGCT and two (4%) of non-TGCT-related reasons (second carcinoma and intracranial haemorrhage).

### Pgp, MRP1, BCRP, and LRP expression in TGCT at diagnosis

The results of immunohistochemistry are summarised in Table 3

**Table 3:** Pgp, MRP1, BCRP, and LRP expressions in testicular germ-cell tumours at diagnosis

**Category**	**Strong^a^**	**Intermediate^a^**	**Negative^a^**
Pgp	15 (27%)	23 (41%)	18 (32%)
MRP1	30 (54%)	21 (37%)	5 (9%)
BCRP	48 (86%)	4 (7%)	4 (7%)
LRP	8 (14%)	16 (29%)	32 (57%)

a Strong: strong staining in >10% of tumour cells; intermediate: positive staining in ⩽10% of tumour cells *or* weak and diffuse positive staining; negative: no staining.

**Figure 1:**
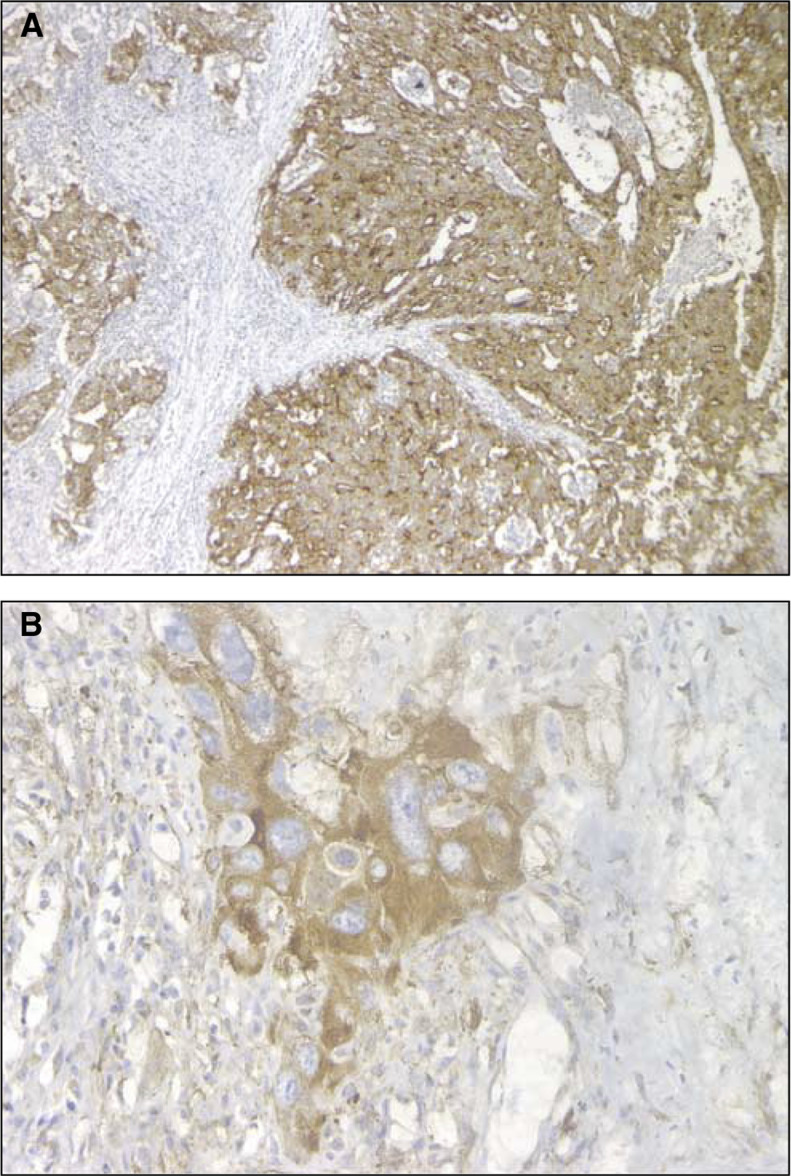
Immunohistochemical staining on paraffin-embedded specimens of testicular germ-cell tumours. (**A**) (× 100) BCRP strong positive embryonal carcinoma stained with MAb BXP-21. Note the strong diffuse membranous and cytoplasmic staining pattern. (**B**) (× 200) LRP strong positive choriocarcinoma stained with MAb LRP. Note the strong granular cytoplasmic pattern in syncytiotrophoblastic cell.

. Fifteen (27%) and 23 (41%) of the 56 specimens showed strong and intermediate expression of Pgp, respectively. The staining pattern was diffuse and homogeneous, cytoplasmic and occasionally membranous. Thirty (54%) and 21 (37%) of the 56 cases displayed strong and intermediate expression of MRP1, respectively. Immunostaining was rather heterogeneous. MRP1 was detected at the cytoplasm, although nuclear and scarce membranous expressions were also found. For both Pgp and MRP1, the intermediate category was composed mainly of samples with a weak expression in over 10% of tumour cells (20 out of 23 and 17 out of 21, respectively). Forty-eight (86%) and four (7%) of the 56 cases displayed, respectively, strong and intermediate expression of BCRP, with a mixed staining pattern, membranous (particularly among EC and YS elements) and cytoplasmic (in syncytiotrophoblasts). BCRP staining intensity was mostly strong and rather homogeneous as shown in Figure 1A

, except for YS elements (usually positive in areas with epithelial differentiation and negative where mesenchymal). Eight (14%) and 16 (29%) of the 56 cases displayed, respectively, strong and intermediate expression of LRP, with a typical granular cytoplasmic pattern as shown in Figure 1B (Izquierdo *et al*, 1996b). Intensity was mostly strong and heterogeneous, with the exception of syncytiotrophoblastic areas, which showed homogeneous staining. Remarkably, 15 of 16 samples in the intermediate category had less than 10% intense positive cells. In eight of these 15, one histological subtype (six CC and two immature teratomas) with a minor overall representation (⩽10%) was found universally positive. No staining was observed among negative controls for all four MAbs. No correlation was found between the expressions of the different proteins (data not shown).

### MDR status and established prognostic factors

For the reasons described before (see Patients and Methods), the intermediate immunohistochemical category was grouped either with the negative or the strong category for statistical correlations with other prognostic factors and analysis of outcome. Table 1 summarises these correlations. Tumours with strong expression of Pgp were more frequently observed in patients with AFP levels <1000 ng ml^−1^ (*P*=0.026). Tumours with strong expression of MRP1 correlated with LDH levels >1.5 upper normal threshold (*P*=0.014). No association could be identified for BCRP. Tumours with strong or intermediate expression of LRP associated with *β*-hCG levels >5000 IU/l^−1^ (*P*=0.003) and stage IV (*P*=0.029) according to the Royal Marsden Hospital (RMH) Classification (Peckham *et al*, 1979).

### MDR status and response to induction chemotherapy

Regardless of the grouping of the intermediate category, no significant association was observed between the expression of Pgp, MRP1, BCRP, and LRP and the response to induction chemotherapy (Table 4

**Table 4:** Expression of Pgp, MRP1, BCRP, and LRP and response to induction chemotherapy (*n*=55 evaluable patients)

	***n***	**CR (%)**	**CR-S + IR (%)**	***P*^a^**
*Pgp*^b^				
+	14	12(86)	2(14)	0.265
−	41	39(95)	2(5)	
*MRP1*^b^				
+	29	26(90)	3(10)	0.613
−	26	25(96)	1(4)	
*BCRP*^b^				
+	47	44(94)	3(6)	0.477
−	8	7(87.5)	1(12.5)	
*LRP*^b^				
+	23	21(91)	2(9)	0.999
−	32	30(94)	2(6)	

CR=complete response to chemotherapy, CR-S=complete response to chemotherapy and surgery, IR=incomplete response (see Patients and Methods).

a Fisher's exact test.

b Pgp+, MRP1+, and BCRP+ represent immunohistochemical category strong; Pgp-, MRP1-, and BCRP- represent the grouping of intermediate and negative categories. The grouping of strong and intermediate categories resulted also in not significant correlations (data not shown) (see Patients and Methods, and Results). LRP+ represents the grouping of categories intermediate and strong; LRP- represents negative category. The grouping of intermediate and negative categories resulted also in not significant correlations (data not shown). For LRP grouping of categories intermediate and strong is shown due to the prognostic value on PFS and OS (see Patients and Methods, and Results).

).

### MDR status and clinical outcome

**Figure 2:**
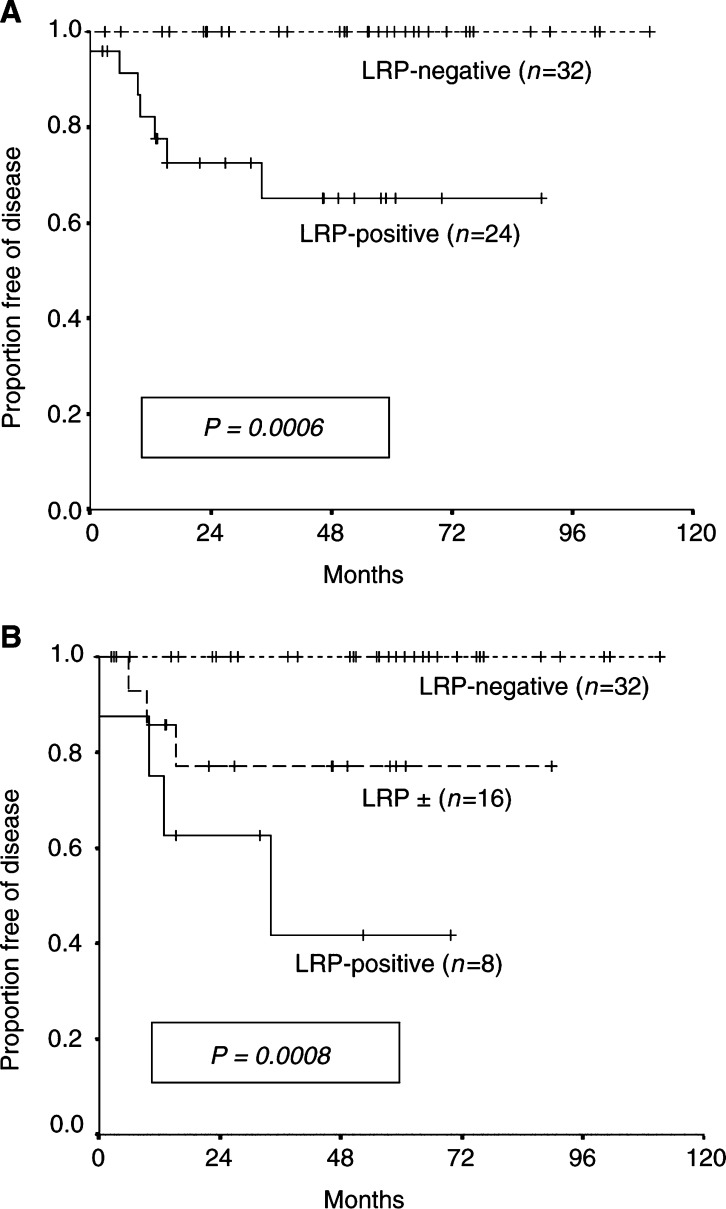
Progression-free survival according to LRP expression previous to treatment in 56 patients with advanced testicular germ-cell tumours. (**A**) Immunohistochemical categories strong and intermediate (grouped) *vs* negative. (**B**) Considering the three original immunostaining categories used for scoring (see Patients and Methods, and Results).

**Figure 3:**
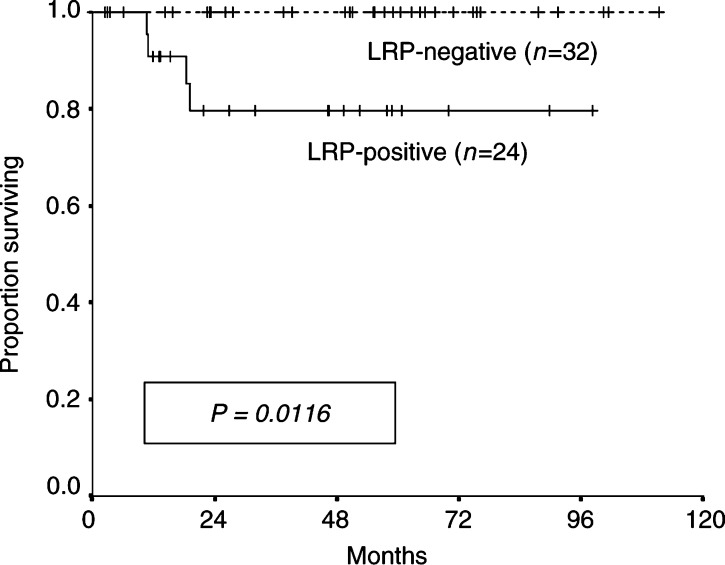
Overall survival according to LRP expression previous to treatment in 56 patients with advanced testicular germ-cell tumours. Immunohistochemical categories: strong and intermediate (grouped) *vs* negative.

Three-year PFS for all 56 patients included in the survival analysis was 85.3% (95% CI, 75.2–95.3%), and 3-year OS 91.4% (83.9–99.4%). With seven relapses and four cancer-related deaths, median PFS and OS have not been reached. Regardless of the grouping of the intermediate category, no association was observed for Pgp, MRP1, and BCRP expressions either with PFS (strong *vs* intermediate/negative: *P*=0.307, 0.284, and 0.284, respectively) or OS (strong *vs* intermediate/negative: *P*=0.932, 0.98, and 0.053, respectively). For all three MDR proteins, the grouping of the strong plus intermediate category resulted in more unbalanced arms (very few patients in the negative category) and in lack of significant correlations (data not shown). In contrast, patients whose tumours showed intermediate or strong expressions of LRP had significantly shorter PFS (*P*=0.0006; Figure 2A

) and OS (*P* = 0.0116; Figure 3

) than LRP-negative patients. When considering the three original immunostaining categories separately, log-rank statistics for PFS and OS were *P*=0.0008 and 0.035, respectively (Figure 2B). The grouping of the intermediate and strong categories discriminated better the outcome according to LRP expression. By univariate analysis, LRP was the strongest adverse prognostic marker for PFS (*P*=0.0006) and OS (*P*=0.0116), followed by *β*-hCG level >5000 IU l^−1^ previous to induction chemotherapy (*P*=0.039 for PFS, and *P*=0.059 for OS). Risk stratification according to the IGCCC (intermediate-poor *vs* good) or RMH (stage IV *vs* IM–II – III) did not achieve prognostic significance either for PFS (*P*=0.07 and 0.481, respectively) or OS (*P*=0.138 and 0.38, respectively) in this relatively small set of patients. As no recurrences or cancer-related deaths (i.e. events) were accounted among LRP-negative patients, no multivariate Cox regression analysis could be performed. Instead, we tested survival distributions for LRP adjusting for statistically related variables performing a stratified Mantel–Haenszel test. LRP remained significant after adjusting for *β*-hCG level (*P*=0.0029 for PFS, and *P*=0.038 for OS), and RMH stage (*P*=0.001 for PFS, and *P*=0.02 for OS).

## DISCUSSION

The increasing need of new markers for prediction of response to chemotherapy and long-term PFS (the most relevant end point for TGCT patients in prognostic factor analyses), and the scarce data concerning the expression and significance of MDR-related proteins in TGCT, prompted us to initiate this study.

The most remarkable finding in our series of advanced TGCT was that LRP immunoreactivity in pretreatment tumour cells was prognostic of the clinical outcome. LRP immunostaining, at any level, was detected in 43% of the patients, consistent with the 50% previously reported in a short series with only 12 specimens (Izquierdo *et al*, 1996b). LRP is the human major protein of cellular organelles known as vaults (Scheffer *et al*, 1995; for review, see Scheffer *et al*, 2000b), ribonucleoprotein particles with a peculiar ovoid structure that is highly conserved among various species. Vaults have been localised in the cytoplasm but also in the nuclear membrane, probably at the nuclear pore complex (Chugani *et al*, 1993). They have been found broadly distributed in normal human tissues and in tumours (Izquierdo *et al*, 1996b). Although vault function is yet undetermined, they are thought to mediate vesicular and nucleo-cytoplasmic trafficking and transport processes of various substrates including cytostatics (Chugani *et al*, 1993; Kitazono *et al*, 1999). The relevance of LRP as a constituent of the whole vault particle in the prediction of a MDR phenotype is well documented *in vitro* in numerous human cancer cell lines (Scheper *et al*, 1993; Izquierdo *et al*, 1996a; Laurençot *et al*, 1997; Kickhoefer *et al*, 1998). Kitazono *et al* (1999) recently provided the first evidence favouring a causal relation between vaults and drug resistance. Moreover, basal LRP expression has been found indicative for cisplatin and carboplatin resistance (nonclassical MDR-related drugs) in the NCI panel of 61 unselected human tumour cell lines used for drug screening (Izquierdo *et al*, 1996a), and in nonsmall-cell lung cancer cell lines (Berger *et al*, 2000).

In our study, any extent of LRP immunoreactivity including strong expression in a minority of cancer cells was associated with an unfavourable prognosis even after individual log-rank adjustments for statistically related prognostic factors. The strong biological and clinical rationale supporting the association of LRP with resistance to a broad spectrum of anticancer drugs, including cisplatin, and the fact that the two correlations found go in the same direction (LRP expression correlates with both shorter PFS and OS), makes unlikely that the positive analyses are due to multiple subset analysis. The frequent presentation of TGCT as mixed histologies and the potential significance for prognosis of a minor component in them make this a unique entity. Our finding adds to several clinical studies reporting overexpression of LRP at diagnosis as an independent predictor for clinical outcome and/or chemotherapy failure, for example, in acute myeloid leukaemia (List *et al*, 1996; Borg *et al*, 1998; Filipits *et al*, 1998,^2000^), multiple myeloma (Raaijmakers *et al*, 1998; Filipits *et al*, 1999), stage III–IV ovarian carcinoma (Izquierdo *et al*, 1995), and locally advanced bladder cancer (Diestra *et al*, 2001). Other studies have failed to show prognostic values of LRP immunoreactivity in acute leukaemia (Damiani *et al*, 1998; Leith *et al*, 1999) and ovarian carcinoma (Arts *et al*, 1999). Despite the prognostic value of LRP in this study, no relation was observed between LRP expression and response to platinum-based chemotherapy. Whether this lack of correlation was because of the very high rate of CR observed (93%, otherwise expected in TGCT) in our relatively small series of patients remains uncertain. As an alternative explanation, LRP may be a marker of biological aggressiveness in TGCT. This is suggested not only by the fact of poorer clinical outcomes for patients with LRP-expressing tumours, but also by the observed correlation between LRP and well-known factors conferring bad prognosis such as high *β*-hCG levels and visceral metastatic disease. Baldini *et al* (1995) found a similar conclusion for Pgp in high-grade osteosarcomas. They reported a strong correlation between the presence of increased levels of Pgp at diagnosis and bad prognosis that was unrelated to response to chemotherapy. We cannot exclude a potential influence of the histological subtype CC over the observed LRP value for prognosis in our series, as all of the CC elements stained for LRP (and *β*-hCG is elaborated by syncytiotrophoblastic components in CC). However, most prognostic classifications have failed to identify any particular histology as an independent variable for outcome (Mead *et al*, 1992). More detailed studies on the relation of LRP with clinicopathological parameters in TGCT are therefore warranted.

Pgp expression was observed in 27% of tumours, in agreement with previous reports (35%, Katagiri *et al*, 1993; 33%, Eid *et al*, 1998). In contrast to the report by Eid *et al* (1998), we found no association between Pgp expression and nonseminomatous histology or advanced stages of disease. This discrepancy can be attributed to our small number of seminomas and the use of different methodologies for Pgp detection. In agreement with the previous study, Pgp expression did not predict response to chemotherapy and was not related to survival. Neither *in vitro* nor clinical data suggest a relation between Pgp expression and resistance to cisplatin. However, the incorporation of etoposide, a substrate for Pgp (Germann, 1996), into combination chemotherapy regimens has improved response rates and long-term survival of poor-risk TGCT patients (Bosl and Motzer, 1997; Sonneveld *et al*, 2001). Whether the expression of Pgp in TGCT influences response to chemotherapy and outcome after regimens containing etoposide needs to be investigated in a much larger group of patients primarily treated with this drug.

There is only one previous study concerning MRP1 expression in TGCT (Eid *et al*, 2000). We report MRP1 overexpression in 54% TGCT, whereas Eid *et al* (2000) reported in 100% using a different MAb but similar criteria for assessment. In our study, MRP1 expression did not predict response to chemotherapy or outcome. All attempts to demonstrate MRP1 as a mediator of cisplatin resistance have failed thus far; although MRP1 is involved in efflux conjugates of drugs to glutathione (one of the cellular thiol compounds involved in resistance to platinum agents; Borst *et al*, 2000). Eid *et al* (2000) reported MRP1 expression in the nucleus, in accordance with our observation, an intriguing binding of unclear significance. They speculated about a possible role for MRP1 unique to TGCT in the intracellular redistribution of drugs. However, we cannot exclude this finding being a staining artefact.

Recently, the novel ATP-dependent transporter BCRP was identified in mitoxantrone-selected MDR cell lines not expressing Pgp or MRP1 (Allikmets *et al*, 1998; Doyle *et al*, 1998). Thus far, BCRP has been described in cancer cell lines of different histogenetic origin (Scheffer *et al*, 2000a). Additionally, expression of BCRP has been reported in normal human tissues (Maliepaard *et al*, 2001), in a small panel of human tumour samples including two seminoma specimens (both considered BCRP-negative; Scheffer *et al*, 2000a), and in human breast carcinoma (Kanzaki *et al*, 2001) and acute leukaemia (Ross *et al*, 2000). We report BCRP overexpression in 86% of TGCT, particularly in EC and in syncytiotrophoblasts in CC. The high expression rate of BCRP in placental syncytiotrophoblast (Maliepaard *et al*, 2001) is therefore retained in its malignant counterpart. BCRP did not predict response to chemotherapy and was not related to survival, consistent with its wide expression in TGCT and the limited role in this disease of the cytostatics to which BCRP confers resistance. In particular, BCRP is not involved in resistance to cisplatin (Allikmets *et al*, 1998; Doyle *et al*, 1998; Maliepaard *et al*, 1999).

In conclusion, our data suggest that the expression of LRP at the time of diagnosis of a metastatic TGCT may identify a patient population with a tendency to progress despite chemotherapy. The adverse prognostic value of LRP may be important because it may influence early selection of TGCT patients for novel therapeutic strategies. Moreover, the search for pharmacological agents capable of affecting vault function might also help in optimising treatment protocols in the future. However, because of the limited number of patients in this study, our results must be interpreted with caution. Whether LRP will increase the predictive power of current clinically oriented prognostic models remains to be determined in larger, prospective studies. The intriguing hypothesis derived from our study may prompt the initiation of such studies by institutions or collaborative groups capable of recruiting a large number of cases of this relatively rare type of cancer. We strongly encourage confirmation or rebuttal of our findings (especially the zero cell) by others.
